# The relationship between intrinsic capacity and sarcopenia in middle-aged and older Chinese populations: the mediating influence of a novel nutritional index

**DOI:** 10.3389/fpubh.2025.1605158

**Published:** 2025-05-21

**Authors:** Hong Ding, Changqing Li, Xiaojiang Zhao

**Affiliations:** Department of Physical Education and Arts, Bengbu Medical University, Bengbu, China

**Keywords:** intrinsic capacity, nutritional index, sarcopenia, CHARLS, cross-sectional study

## Abstract

**Background:**

Sarcopenia poses a major global health issue, with intrinsic capacity (IC) linked to its risk. This study examined the link between IC and sarcopenia in middle-aged and older Chinese individuals, focusing on the mediating role of triglycerides-total cholesterol-body weight index (TCBI), a new easy-to-calculate nutritional indicator.

**Methods:**

The investigation utilized 2015 data from the China Health and Retirement Longitudinal Study (CHARLS), focusing on individuals aged 45 years or older. Sarcopenia was evaluated using the 2019 guidelines from the Asian Sarcopenia Working Group. After adjusting for various confounders, we employed multiple logistic regression to explore the link between IC, TCBI, and sarcopenia, and used a mediation model to evaluate TCBI’s role in the IC-sarcopenia. Subgroup analysis examined the heterogeneity among various groups.

**Results:**

This research encompassed a cohort of 6,554 individuals, displaying an average age of 59.4 ± 9.1 years, comprising 5,758 non-sarcopenia and 796 sarcopenia individuals. Mean IC was 5.1 ± 1.0 for non-sarcopenia group and 4.4 ± 1.2 for sarcopenia group. In the non-sarcopenia group, the median TCBI was 1446.4, and the interquartile range (IQR) was 922.3–2283.4. In the sarcopenia group, the median TCBI was 841.6, and the IQR was 584.9–1304.1. Significant differences in IC and TCBI were observed between the non-sarcopenia and sarcopenia groups (*p* < 0.001). Following rigorous adjustment for all covariates, IC was inversely associated with sarcopenia, and the risk of sarcopenia decreased by 15% for each unit increase in IC (OR = 0.85, 95%CI: 0.76–0.95, *p* = 0.004). Higher TCBI was associated with a 28% decrease in sarcopenia risk per unit increase (OR = 0.72, 95% CI: 0.58–0.90, *p* = 0.004). TCBI’s indirect effect on the IC-sarcopenia link was −4.91 × 10^−3^ (*p* < 0.001), explaining 12.29% of the total effect variation.

**Conclusion:**

The study found that IC is negatively correlated with sarcopenia, while TCBI is negatively correlated with sarcopenia, and TCBI mediates the relationship between IC and sarcopenia.

## Introduction

Demographic aging is progressing at an unprecedented rate worldwide, driven by extended longevity and reduced birth rates (1). Projections indicate that this demographic shift will substantially elevate the number of individuals requiring care support in coming decades (2). Among aging-related conditions, sarcopenia—a progressive loss of muscle mass and strength—represents a prevalent contributor to care dependency challenges (3). Addressing this condition is critical for enhancing intrinsic capacity (IC), a foundational concept enabling transitions from disease-focused healthcare models to function-oriented frameworks, thereby advancing objectives of healthy aging (4, 5).

Sarcopenia, a degenerative musculoskeletal condition linked to aging, involves the progressive reduction of muscle mass, diminished strength, and impaired physical function (4). As demographic shifts toward older age groups intensify globally, sarcopenia prevalence is increasing steadily, affecting 10–27% of populations across varied geographical contexts (6). Extensive research links this condition to elevated risks of functional impairment, nutritional deficiencies, fall-related injuries, and premature death (7, 8), contributing to escalating societal, individual, and financial pressures projected for future generations (9). Emerging data indicate that structured exercise regimens, nutritional optimization, and activity modifications can markedly enhance muscular integrity, power, and functional capacity (10, 11). Given its high prevalence and associations with both acute and chronic health complications, identifying modifiable risk factors and elucidating mechanisms to mitigate sarcopenia’s development remain critical priorities for public health initiatives.

In pursuit of “healthy aging” objectives, the World Health Organization (WHO) introduced the framework of IC in its 2015 Global Report on Aging and Health. IC represents the composite of an individual’s cognitive and physiological reserves, redirecting geriatric care priorities from pathology management to functional optimization. This capacity typically diminishes with advancing age (12). Empirical studies have established robust correlations between IC deficits and heightened risks of frailty (13), fall-related injuries (14), and premature mortality (15)—outcomes exacerbated by sarcopenia’s progression (16). IC is operationalized through standardized metrics evaluating five interconnected domains: locomotion, cognitive, psychology, sensory acuity (vision and hearing), and vitality (5). Notably, deterioration of IC parameters was associated with an increased risk of sarcopenia (17–22). Scholars advocate that systematic tracking of IC may serve as a predictive indicator for functional decline or negative health events, enabling timely preventative strategies (23). However, gaps persist in understanding the biological pathways linking IC degradation to sarcopenia pathogenesis, warranting deeper mechanistic investigation.

The widespread implementation of existing nutrition assessment tools in clinical practice has been hindered by their complexity. To overcome this limitation, Doi et al. proposed a novel and readily calculable index, termed the Triglycerides (TG), Total Cholesterol (TC), and Body Weight (BW) Index (TCBI) (24).


$$\text{TCBI}=\frac{\mathrm{TG}\;(\mathrm{mg}/\mathrm{dL})\times \mathrm{TC}\;(\mathrm{mg}/\mathrm{dL})\times \mathrm{BW}\;(\mathrm{kg})}{1,000}$$

TCBI represents an innovative nutritional index that synthesizes TG, TC, and BW to offer a comprehensive assessment of metabolic health. Compared with traditional lipid indicators, such as low-density lipoprotein (LDL), high-density lipoprotein (HDL), and the ratio of triglycerides to HDL (TG/HDL), TCBI offers the advantage of integrating lipid profiles with body composition (body weight). It captures the synergistic effects of metabolism and nutrition while simplifying risk stratification through a single composite score.

Empirical evidence indicates that a higher TCBI is correlated with enhanced metabolic health, potentially mitigating cognitive decline in middle-aged and older populations (25). TCBI was also inversely associated with the incidence of stroke and stroke-associated pneumonia (SAP) (26, 27). Furthermore, TCBI can serve as a prognostic indicator for coronary heart disease (24), critical illness requiring mechanical circulatory support (MCS) devices (28), and heart failure (29). Moreover, elevated levels of TG and TC, which constitute components of the TCBI, have been linked to depressive symptoms in midlife (30). Depressive symptoms serve as a psychological indicator associated with IC. The evaluation of IC encompasses vitality, which is measured by the body mass index (BMI) and reflects nutritional status (31). There is a positive correlation between BMI and lipid abnormalities, including increased levels of TG and TC (32). Previous studies suggest that TG, TC, and BW may be inversely related to sarcopenia (33–35). However, the relationship between TCBI and sarcopenia remains ambiguous, necessitating further investigation to elucidate the underlying mechanisms.

This cross-sectional study employed data from the 2015 China Health and Retirement Longitudinal Study (CHARLS), a nationally representative survey, to investigate the associations among IC, TCBI, and sarcopenia in individuals aged 45 years and older. Utilizing a mediation analysis framework, the study sought to evaluate whether TCBI functions as a mediating variable in the pathways linking IC to sarcopenia. The analysis quantified both the total effects of IC on sarcopenia and the indirect effects mediated via TCBI, thereby offering insights into potential mechanistic connections between IC, TCBI, and sarcopenia in middle-aged and older adults.

## Study methodology

### Data collection

**Figure 1:**
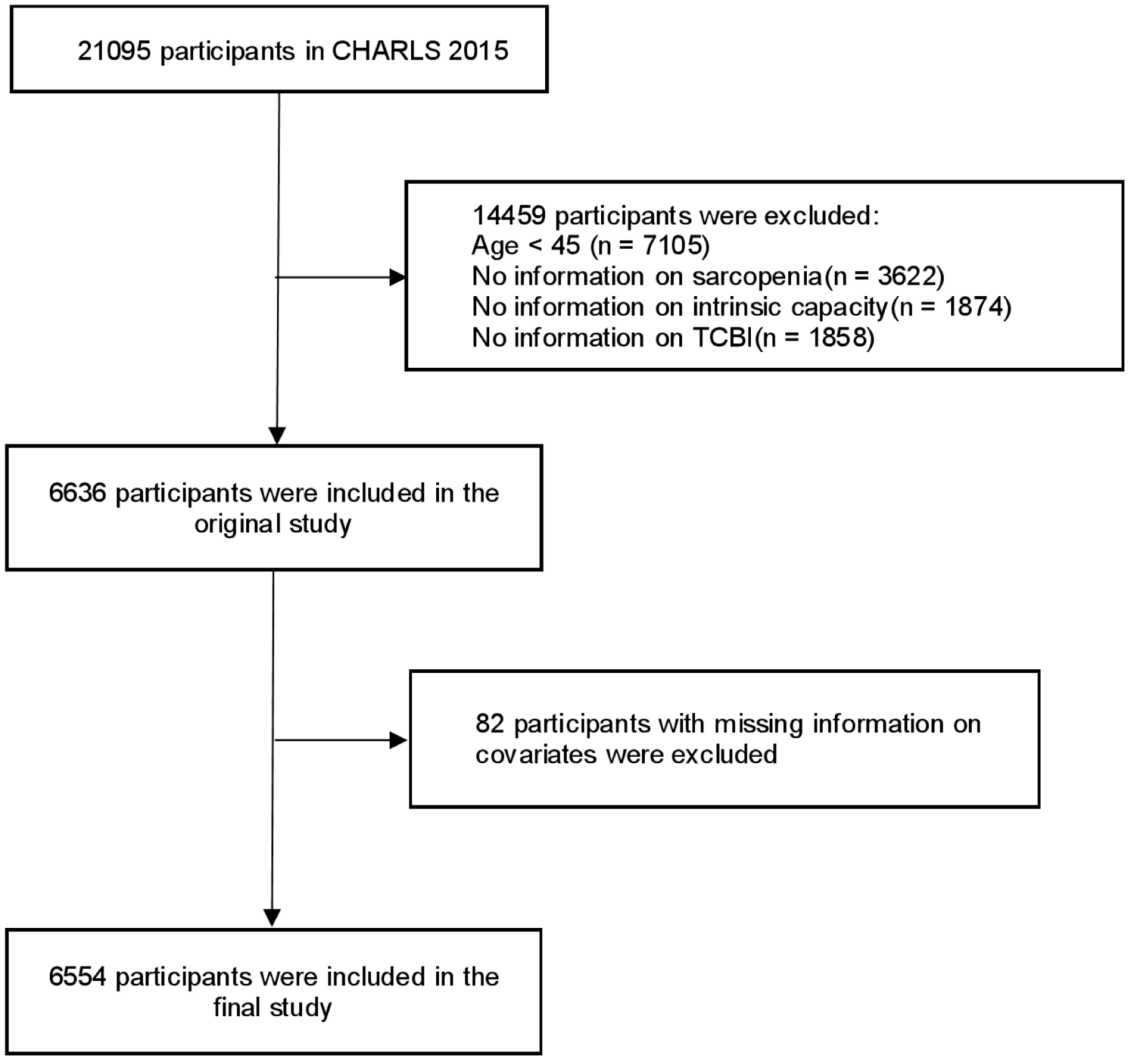
Flowchart of the participants selection process. CHARLS, China health and retirement longitudinal study; TCBI, triglycerides-total cholesterol-body weight index.

This cross-sectional analysis drew upon data from the CHARLS, a population-based longitudinal survey designed to represent adults aged 45 years and older across China. Initiated to compile an extensive repository of health, socioeconomic, and demographic data for aging populations in China, CHARLS employs a multistage probability-proportional-to-size (PPS) sampling methodology across 28 provincial-level administrative units to ensure geographical and sociodemographic diversity. Comprehensive documentation of its stratified sampling design and operational protocols is available in prior publications (36). From the 2015 baseline dataset, which originally comprised 21,095 participants, stringent eligibility criteria were applied to enhance analytical validity. Exclusion criteria removed the following groups: individuals aged <45 years (*n* = 7,105), those with incomplete sarcopenia diagnostic assessments (*n* = 3,622), participants lacking IC measurements (*n* = 1,874), cases without TCBI data (*n* = 1,858), and subjects with missing covariate information (*n* = 82). After applying these filters, the final analytical cohort included 6,554 eligible individuals. A detailed flowchart outlining participant selection and attrition is presented in Figure 1.

### Assessments

#### Sarcopenia

Per the 2019 Asian Working Group for Sarcopenia (AWGS2019) criteria, individuals demonstrating normal chair stand performance alongside preserved muscular strength are excluded from sarcopenia classification (37). Participants not fulfilling all diagnostic thresholds for sarcopenia may be designated as “possible sarcopenia” if they exhibit diminished muscular strength or compromised functional capacity, as evaluated via chair-rising tests. A confirmed sarcopenia diagnosis, however, requires identification of low muscle quantity co-occurring with either weakened strength or diminished functional capacity.

Skeletal muscle strength was assessed through handgrip strength (HGS) measurements. Trained examiners performed these assessments utilizing a calibrated TM WL-1000 dynamometer, configured to measure in kilograms. The mean of the peak values obtained from two trials was calculated to determine muscle strength. A reduction in muscle strength is defined by HGS values below 18 kg for women and below 28 kg for men (37).

Physical performance was assessed utilizing the Short Physical Performance Battery (SPPB), in conjunction with chair stand and gait speed assessments. Functional impairment in physical performance was operationalized as either a gait speed below 1.0 m/s, a time exceeding 12 s to complete five consecutive chair stands, or a composite score ≤9 on the SPPB (37).

Appendicular skeletal muscle mass (ASM), a critical indicator of muscle quantity, was assessed utilizing validated anthropometric predictive equations (38, 39). Empirical research has consistently shown a robust concordance between ASM values obtained from these equations and those measured by dual-energy X-ray absorptiometry (DXA), which is considered the gold standard for body composition analysis. Importantly, this methodological concordance has been specifically observed within Chinese population cohorts (40).

$$\begin{array}{l} \mathrm{ASM}=0.193\times \text{weight}\;(\mathrm{kg})+0.107\times \text{height}\;(\mathrm{cm})-4.157\\ \times \mathrm{sex}\;(1=\text{male},2=\text{female})-0.037\times \mathrm{age}\;(\text{years})-2.631 \end{array}$$

Anthropometric measurements were conducted utilizing standardized instruments: height was measured with a Seca TM213 stadiometer, and weight was assessed using an HN-286 scale. The height-adjusted muscle mass (ASM/Ht^2^) was determined by dividing the appendicular skeletal muscle mass (ASM) by the square of the height in meters. A reduction in muscle mass was identified based on sex-specific thresholds, established at the 20th percentile of ASM/Ht^2^ values within the cohort, defined as ≤6.79 kg/m^2^ for males and ≤4.90 kg/m^2^ for females (38, 39).

#### IC

IC was operationalized through five functional domains: vitality, cognition, locomotion, psychology, and sensory (encompassing both vision and hearing). Each domain was dichotomously coded (1 = preserved functionality, 0 = identifiable deficits), yielding an aggregate score between 0 and 6. Elevated scores reflected enhanced composite capacity, with a threshold of ≤5 demarcating compromised IC, as validated in prior studies (41, 42).

Locomotion: Successfully completing the sit-to-stand test five times independently within 14 s or less was assigned a score of 1 point, whereas completion in more than 14 s was assigned a score of 0 points (43).

Vitality: Vitality was operationalized through BMI assessment, with classification criteria adapted from the Malnutrition Universal Screening Tool (MUST). Individuals were stratified into two categories: those with BMI < 18.5 kg/m^2^ received a score of 0 (indicating nutritional risk), while a BMI ≥ 18.5 kg/m^2^ was designated as 1 (no nutritional risk) (44).

Sensor: (1) Hearing: Auditory capacity was assessed through self-reporting using a single-item query: “How is your hearing?” A dichotomous scoring system was applied, where participants reporting “poor” hearing received a score of 0 point, whereas ratings of “fair,” “good,” “very good,” or “excellent” were coded as 1 point. (2) Vision: Participants were queried with the questions, “How well do you perceive distant objects?” and “How well do you perceive nearby objects?” Participants reporting “fair,” “good,” “very good,” or “excellent” for both questions received a score of 1 point. In contrast, any “poor” response for either question yielded a score of 0 points.

Cognition: Cognitive function was evaluated using the Telephone Interview of Cognitive Status (TICS) (45), a validated tool integrating memory and mental status evaluations. The diagnostic threshold was defined as the mean value minus one standard deviation (SD). Memory retention was tested via immediate and delayed recall tasks involving 10 unrelated terms. Participants were tasked with recalling these terms after intervals of approximately two and four minutes. A maximum score of 20 points was allocated to individuals who succeeded in accurately retrieving all 20 terms. Mental status was evaluated by awarding five points for correct orientation (identifying the current day, month, year, day of the week, and season), five points for accurate calculation ability (subtracting seven from 100 consecutively five times), and one point for visuoconstruction skills (reproducing a picture of two five-pointed stars as demonstrated by the interviewers). The total possible score for mental status was 11 points. Cognitive function was assigned a score of one if neither memory nor mental status showed decline; otherwise, a score of zero was assigned.

Psychology: Per the Center for Epidemiologic Studies Depression Scale (CES-D) (46), participants scoring <12 were classified as having no significant depressive symptoms (assigned 1 point), while scores ≥12 indicated clinically relevant depressive symptoms (assigned 0 points).

#### TCBI

Venous blood samples collected from CHARLS participants through venipuncture were centrifuged and subsequently transported to the Chinese Center for Disease Control and Prevention (China CDC) in Beijing for further processing. The samples were stored at −80°C before being analyzed at the Clinical Laboratory Center of Capital Medical University (CMU). Lipid profiles, including TC, low-density lipoprotein cholesterol (LDL-C), high-density lipoprotein cholesterol (HDL-C), and TG, were assessed using enzymatic colorimetric techniques. The TCBI was calculated using the formula:


$$\text{TCBI}=\frac{\mathrm{TG}\;(\mathrm{mg}/\mathrm{dL})\times \mathrm{TC}\;(\mathrm{mg}/\mathrm{dL})\times \mathrm{BW}\;(\mathrm{kg})}{1,000}$$

High-sensitivity C-reactive protein (hsCRP) levels were quantified using immunoturbidimetric assays, a standardized laboratory technique for assessing inflammatory biomarkers.

### Control variables

The baseline investigation assessed a comprehensive array of demographic and health-related variables. These variables included age, sex, geographic residency (categorized as urban or rural), and marital status, which was further classified into categories such as married and cohabiting with a spouse, married but not cohabiting with a spouse, single, divorced, and widowed. Additional covariates comprised the number of chronic conditions (categorized as none, single, or multiple), smoking status (classified as smoker or non-smoker), alcohol consumption status (categorized as non-drinker, drinking <1/month, or drinking ≥1/month), and highest educational attainment (dichotomized into ≤elementary school and ≥middle school). Furthermore, sleep duration and daytime napping duration were evaluated. The prevalence of chronic diseases was determined based on self-reported diagnoses of fourteen non-communicable conditions, including hypertension, diabetes, dyslipidemia, chronic pulmonary disease, hepatic disorders, renal disease, cardiovascular events (such as myocardial infarction and stroke), malignancies, arthritis, asthma, gastrointestinal ailments, cognitive impairment, mental health conditions, and musculoskeletal disorders. Anthropometric data—height and weight—were collected using standardized techniques to compute BMI. Participants were stratified into four weight categories per WHO guidelines: underweight (BMI < 18.5 kg/m^2^), normal (18.5–24.9), overweight (25–29.9), and obese (≥30).

### Statistical analysis

Sample attributes were analyzed through descriptive statistical methods. Continuous measures were summarized as mean ± SD alongside median and interquartile ranges (IQR). Categorical data were expressed as counts and proportions. A Shapiro–Wilk test assessed the normality of continuous variables, and a natural logarithmic transformation was applied to IC and TCBI. Intervariable relationships were evaluated using Spearman’s correlation coefficients. To investigate TCBI as a mediator between IC and sarcopenia, the study employed the Baron-Kenny causal steps approach (47). Analyses were conducted through a sequential linear regression framework: (1) examining IC’s association with TCBI, (2) assessing IC’s direct relationship with sarcopenia, and (3) evaluating mediation by incorporating TCBI into the IC-sarcopenia model. Association magnitudes were quantified through odds ratios (ORs), with total and indirect effects estimated via nonparametric bootstrap resampling (1,000 iterations) (48). Stratified subgroup analyses explored effect heterogeneity across predefined demographic and health-related categories. Owing to the number of statistical tests we performed, a Bonferroni correction for multiple testing. Analyses adjusted for sociodemographic factors (age, sex, education level, marital status, geographic residence), health behaviors (smoking, alcohol use, sleep duration, daytime napping), and clinical parameters (BMI, number of chronic conditions). Mediation significance was determined using 95% bias-corrected accelerated confidence intervals (CIs) excluding null values. All computations were performed in R (v4.3.2), with statistical significance defined at *α* = 0.05 (two-tailed).

## Result

### Baseline characteristics of the study participants

The study cohort was divided into non-sarcopenia and sarcopenia subgroups, with their demographic and clinical characteristics detailed in Table 1. The analysis included 6,554 participants, with an average age of 59.4 ± 9.1 years. Of these, 3,085 were female (47.1%) and 3,469 were male (52.9%). There were 5,758 in the non-sarcopenia group and 796 in the sarcopenia group. The mean age of non-sarcopenia group was 58.2 ± 8.6 years, and 2,549 (44.3%) were women and 3,209 (55.7%) were men. The mean age of sarcopenia group was 68.0 ± 7.5 years. There were 536 females (67.3%) and 260 males (32.7%). A significant proportion of participants lived in rural areas (58%) and were married or cohabiting with their spouses (85.6%). Significant variations emerged across demographic and health-related variables—including age, gender distribution, geographic residence, educational attainment, marital status, lifestyle factors (tobacco use, alcohol intake), sleep patterns (sleep duration, daytime napping), BMI, and chronic disease prevalence—with statistical significance maintained across all measures (*p* < 0.001). Notably, the mean IC for the non-sarcopenia group was 5.1 ± 1.0, which was significantly different from the mean IC of 4.4 ± 1.2 observed in the sarcopenia group (*p* < 0.001). Similarly, the median TCBI in the non-sarcopenia group was 1355.9 (IQR 922.3 to 2283.4), which significantly differed from the median TCBI of 841.6 (IQR 584.9 to 1304.1) in the sarcopenia group (*p* < 0.001).

**Table 1:** Characteristics of the study participants.

Variables	Overall	Non-sarcopenia	Sarcopenia	*p*-value
	*n* = 6,554	*n* = 5,758	*n* = 796	
Age, Mean ± SD	59.4 ± 9.1	58.2 ± 8.6	68.0 ± 7.5	<0.001
Sex, *n* (%)				<0.001
Female	3,085 (47.1)	2,549 (44.3)	536 (67.3)	
Male	3,469 (52.9)	3,209 (55.7)	260 (32.7)	
Residence, *n* (%)				<0.001
Rural	3,804 (58.0)	3,247 (56.4)	557 (70)	
Urban	2,750 (42.0)	2,511 (43.6)	239 (30)	
Marital status, *n* (%)				<0.001
Married and living with a spouse	5,613 (85.6)	5,008 (87)	605 (76)	
Married but living without a spouse	276 (4.2)	258 (4.5)	18 (2.3)	
Single, divorced, and windowed	665 (10.1)	492 (8.5)	173 (21.7)	
Education Status, *n* (%)				<0.001
Elementary school or below	4,065 (62.0)	3,434 (59.6)	631 (79.3)	
Middle school or above	2,489 (38.0)	2,324 (40.4)	165 (20.7)	
Smoking status, *n* (%)				<0.001
Smoker	3,432 (52.4)	2,941 (51.1)	491 (61.7)	
Non-smoker	3,122 (47.6)	2,817 (48.9)	305 (38.3)	
Drinking status, *n* (%)				<0.001
Drink but less than once a month	637 (9.7)	569 (9.9)	68 (8.5)	
Drink more than once a month	1935 (29.5)	1781 (30.9)	154 (19.3)	
Non-drinker	3,982 (60.8)	3,408 (59.2)	574 (72.1)	
Sleep duration (Hrs.), Mean ± SD	6.5 ± 1.8	6.5 ± 1.7	6.2 ± 2.1	<0.001
Daytime napping duration (Min), Mean ± SD	41.0 ± 44.8	41.9 ± 44.6	34.9 ± 45.6	<0.001
BMI group, *n* (%)				<0.001
Normal	204 (3.2)	30 (0.5)	174 (21.9)	
Obesity	3,596 (55.8)	2,985 (52.8)	611 (77)	
Overweight	2,217 (34.4)	2,209 (39.1)	8 (1)	
Underweight	427 (6.6)	426 (7.5)	1 (0.1)	
Number of chronic conditions, *n* (%)				<0.001
0	1952 (29.8)	1764 (30.6)	188 (23.6)	
1	1,508 (23.0)	1,320 (22.9)	188 (23.6)	
≥2	3,094 (47.2)	2,674 (46.4)	420 (52.8)	
IC, Mean ± SD				<0.001
	5.0 (1.1)	5.1 (1.0)	4.4 (1.2)	
TCBI, Median (IQR)	1355.9 (856.6, 2178.4)	1446.4 (922.3, 2283.4)	841.6 (584.9, 1304.1)	<0.001

BMI, body mass index; Hrs., hours; Min, minutes; TCBI, triglyceride-cholesterol-body weight index.

### Associations of IC and TCBI with sarcopenia

Table 2 displays correlations between IC, TCBI, and sarcopenia. The analysis examined IC’s association with sarcopenia across 6,554 participants. Unadjusted analyses revealed a statistically significant negative link between IC and sarcopenia, yielding an OR of 0.57 (95% CI: 0.53–0.61; *p* < 0.001). After adjusting for covariates in Model 1, the effect size attenuated slightly (OR = 0.75, 95% CI: 0.70–0.81; *p* < 0.001). Further adjustments in Model 2 (OR = 0.75, 95% CI: 0.69–0.81; *p* < 0.001) and Model 3 (OR = 0.85, 95% CI: 0.76–0.95; *p* = 0.004) demonstrated persistent significance. This consistency indicates that IC was consistently a protective factor for sarcopenia despite adjustment for covariates.

**Table 2:** Association of IC and TCBI with sarcopenia.

Variables	No.	Unadjusted		Model 1		Model 2		Model 3	
		OR (95% CI)	*p* value	OR (95% CI)	*p* value	OR (95% CI)	*p* value	OR (95% CI)	*p* value
IC	6,554	0.57 (0.53 ~ 0.61)	<0.001	0.75 (0.70 ~ 0.81)	<0.001	0.75 (0.69 ~ 0.81)	<0.001	0.85 (0.76 ~ 0.95)	0.004
TCBI	6,554	0.27 (0.23 ~ 0.31)	<0.001	0.19 (0.16 ~ 0.23)	0.001	0.19 (0.16 ~ 0.23)	<0.001	0.72 (0.58 ~ 0.90)	0.004

Model 1: adjusted for age, gender, educational level, marital status, and residence. Model 2: adjusted for model 1 + smoking status, drinking status, sleep duration and daytime napping duration. Model 3: adjusted for model 2 + BMI and 14 chronic diseases. OR, odds ratio; 95% CI, 95% confidence interval; TCBI, triglyceride-cholesterol-body weight index.

TCBI demonstrated a significant inverse association with sarcopenia in the analysis (OR = 0.27, 95% CI: 0.23–0.31; *p* < 0.001). This relationship retained statistical significance across sequential adjustments: Model 1 (OR = 0.19, 95% CI: 0.16–0.23; *p* = 0.001), Model 2 (OR = 0.19, 95% CI: 0.16–0.23; *p* < 0.001), and Model 3 (OR = 0.72, 95% CI: 0.58–0.90; *p* = 0.004). After adjusting for covariates, the robustness of the negative association between TCBI and sarcopenia persisted, despite being influenced by BMI and 14 chronic diseases. These findings suggest that TCBI continues to serve as a protective factor against sarcopenia.

In summary, IC was consistently significantly negatively associated with sarcopenia after controlling for relevant variables, but the adjusted effect size fluctuated, indicates that this association may be partially influenced by confounding factors, such as age, gender, educational level, marital status, residence, BMI and 14 chronic diseases, but is not significant. For the association of TCBI with sarcopenia, models 1 to 3 showed that the protective effect of TCBI on sarcopenia was influenced by demographic factors (age, gender, etc.), lifestyle factors (smoking, drinking, sleep), BMI and 14 chronic diseases. The effect size was significantly reduced, but its independent protective effect was still highly significant.

Furthermore, the association analyses between IC and TG, TC, and BW, as well as between TG, TC, BW, and sarcopenia, are detailed in Supplementary Tables 1, 2.

Tables 3, 4 show that no significant modifying factors were found for the association between IC, TCBI and sarcopenia after Bonferroni correction.

**Table 3:** Subgroup analysis of the association between intrinsic capacity and sarcopenia.

Subgroup	n. total	Event (%)	OR (95% CI)	P for interaction
Age				0.07
<65	4,647	266 (5.7)	0.83 (0.68 ~ 1.01)	
≧65	1907	530 (27.8)	0.86 (0.75 ~ 0.99)	
Sex				0.08
Male	3,085	536 (17.4)	0.81 (0.71 ~ 0.93)	
Female	3,469	260 (7.5)	1.06 (0.84 ~ 1.35)	
Residence				0.05
Rural	3,804	557 (14.6)	0.89 (0.78 ~ 1.01)	
Urban	2,750	239 (8.7)	0.73 (0.58 ~ 0.91)	
Marital status				0.84
Married and living with a spouse	5,613	605 (10.8)	0.84 (0.73 ~ 0.97)	
Married but living without a spouse	276	18 (6.5)	0.75 (0.15 ~ 3.84)	
Single, divorced, and windowed	665	173 (26)	0.94 (0.76 ~ 1.16)	
Education status				0.39
Elementary school or below	4,065	631 (15.5)	0.87 (0.77 ~ 0.98)	
Middle school or above	2,489	165 (6.6)	0.84 (0.63 ~ 1.12)	
Smoking status				0.34
Smoker	3,432	491 (14.3)	0.84 (0.74 ~ 0.97)	
Non-smoker	3,122	305 (9.8)	0.91 (0.73 ~ 1.13)	
Drinking status				0.11
Non-drinker	3,982	574 (14.4)	0.84 (0.72 ~ 0.98)	
Drink but less than once a month	637	68 (10.7)	0.64 (0.47 ~ 0.86)	
Drink more than once a month	1935	154 (8)	1.05 (0.79 ~ 1.41)	
BMI group				0.89
Normal	204	174 (85.3)	0.7 (0.44 ~ 1.14)	
Obesity	3,596	611 (17)	0.82 (0.71 ~ 0.95)	
Overweight	2,217	8 (0.4)	1.23 (0.45 ~ 3.4)	
Underweight	427	1 (0.2)	309.56 (0 ~ Inf)	
Number of chronic conditions				0.78
0	1952	188 (9.6)	0.76 (0.56 ~ 1.03)	
1	1,508	188 (12.5)	0.86 (0.65 ~ 1.13)	
≧2	3,094	420 (13.6)	0.89 (0.78 ~ 1.01)	

Adjusted for age, gender, educational level, marital status, smoking status, drinking status, sleep duration daytime napping duration BMI, and 14 chronic diseases. P for interaction <0.0056 was considered significant, as we had to correct our analysis for multiple testing (P for interaction of 0.0056 was calculated as: 0.05 divided by 9). OR, odds ratio; 95% CI, 95% confidence interval; BMI, body mass index; TCBI, triglyceride-cholesterol-body weight index.

**Table 4:** Subgroup analysis of the association between TCBI and sarcopenia.

Subgroup	n. total	Event (%)	OR (95% CI)	P for interaction
Age				0.06
<65	4,647	266 (5.7)	1.07 (0.76 ~ 1.52)	
≧65	1907	530 (27.8)	0.52 (0.38 ~ 0.7)	
Sex				0.25
Male	3,085	536 (17.4)	0.65 (0.5 ~ 0.84)	
Female	3,469	260 (7.5)	0.79 (0.5 ~ 1.27)	
Residence				0.54
Rural	3,804	557 (14.6)	0.69 (0.53 ~ 0.89)	
Urban	2,750	239 (8.7)	0.81 (0.53 ~ 1.24)	
Marital status				0.97
Married and living with a spouse	5,613	605 (10.8)	0.88 (0.67 ~ 1.16)	
Married but living without a spouse	276	18 (6.5)	2.56 (0.08 ~ 83.58)	
Single, divorced, and windowed	665	173 (26)	0.38 (0.24 ~ 0.59)	
Education status				0.53
Elementary school or below	4,065	631 (15.5)	0.66 (0.52 ~ 0.85)	
Middle school or above	2,489	165 (6.6)	0.97 (0.58 ~ 1.63)	
Smoking status				0.03
Smoker	3,432	491 (14.3)	0.7 (0.53 ~ 0.91)	
Non-smoker	3,122	305 (9.8)	0.68 (0.44 ~ 1.06)	
Drinking status				0.56
Non-drinker	3,982	574 (14.4)	0.83 (0.61 ~ 1.13)	
Drink but less than once a month	637	68 (10.7)	0.15 (0.08 ~ 0.29)	
Drink more than once a month	1935	154 (8)	1.08 (0.66 ~ 1.76)	
BMI group				0.25
Normal	204	174 (85.3)	1.4 (0.64 ~ 3.07)	
Obesity	3,596	611 (17)	0.83 (0.62 ~ 1.11)	
Overweight	2,217	8 (0.4)	1.5 (0.22 ~ 10.36)	
Underweight	427	1 (0.2)	291.63 (0 ~ Inf)	
Number of chronic conditions				0.51
0	1952	188 (9.6)	0.96 (0.57 ~ 1.61)	
1	1,508	188 (12.5)	0.85 (0.49 ~ 1.45)	
≧2	3,094	420 (13.6)	0.62 (0.47 ~ 0.83)	

Adjusted for age, gender, educational level, marital status, smoking status, drinking status, sleep duration, daytime napping duration, BMI, and 14 chronic diseases. P for interaction < 0.0056 was considered significant, as we had to correct our analysis for multiple testing (P for interaction of 0.0056 was calculated as: 0.05 divided by 9). OR, odds ratio; 95% CI, 95% confidence interval; BMI, body mass index; TCBI, triglyceride-cholesterol-body weight index.

### TCBI mediated the association between IC and sarcopenia

Table 5 presents the relationships between baseline IC, the TCBI, and sarcopenia. Statistical analyses revealed a significant inverse correlation between IC and sarcopenia (*r* = −0.23, *p* < 0.001). Conversely, a positive association was identified between IC and TCBI (*r* = 0.11, *p* < 0.001). Most strikingly, the relationship between TCBI and sarcopenia demonstrated a stronger inverse correlation (*r* = −0.25, *p* < 0.001), highlighting its more robust predictive capacity.

**Table 5:** Association among IC and TCBI with sarcopenia.

Variables	IC	TCBI	Sarcopenia
Intrinsic capacity	1.00		
TCBI	0.11***	1.00	
Sarcopenia	−0.23***	−0.25***	1.00

*** *p*-value <0.001. TCBI, triglyceride-cholesterol-body weight index.

**Figure 2:**
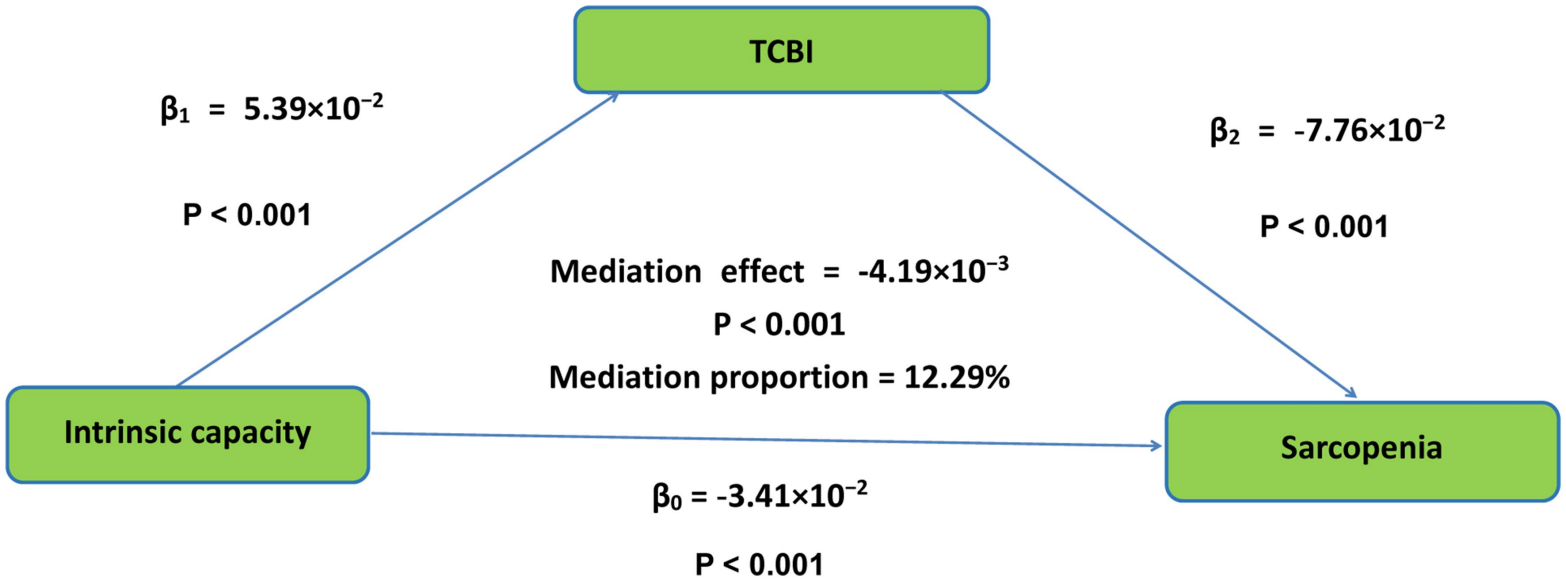
The conceptional framework of the mediation models. *β*0 was the total effect of intrinsic capacity on sarcopenia; *β*1 represents the effect of intrinsic capacity on TCBI; *β*2 represents the effect of TCBI on sarcopenia. The mediation effect was computed as the product of “*β*1” and “*β*2” (*β*1 × *β*2), and the mediation proportion was calculated as the ratio of the mediation effect product to total effects [(*β*1 × *β*2)/*β*0]. TCBI, triglycerides-total cholesterol-body weight index.

Bootstrap analysis revealed the total effect of baseline IC on sarcopenia (*β*₀ = −3.41 × 10^−2^, *p* < 0.001). TCBI was found to significantly mediate the relationship between IC and sarcopenia, with a mediation effect size of −4.91 × 10^−3^ (*p* < 0.001). This mediating pathway accounted for 12.29% of the total effect variation. A visual representation of this mediation pathway is provided in Figure 2.

## Discussion

This study is the first to explore the connection between IC, TCBI, and sarcopenia in middle-aged and older Chinese individuals using 2015 CHARLS data. It found that both IC and TCBI are inversely related to sarcopenia risk, with TCBI mediating the effect of IC deficits on sarcopenia thus supports our original hypothesis.

Biological aging, influenced by inherent mechanisms, leads to decreased IC in sensory, vitality, and psychological areas as people age. This complex process explains the varied functional decline among individuals, affecting more than just physical and cognitive abilities (12). It also includes sensory, vitality, and psychological aspects, which are crucial to IC (49). IC effectively captures the diversity of functional capacity in the older population, regardless of clinical phenotypes (50). Sarcopenia is an age-related condition marked by reduced muscle mass, strength, and physical performance. Managing sarcopenia is crucial for shifting from a disease-centered to a function-centered approach, promoting healthy aging (4, 5). After accounting for confounding factors, a multiple logistic regression analysis showed that each unit increase in IC reduced sarcopenia risk by 15% (OR = 0.85, 95% CI: 0.76–0.95; *p* = 0.004), indicating IC might protect against sarcopenia.

Research suggests that common risk factors, such as inflammation, may influence both cognitive decline and sarcopenia. In particular, inflammation, which involves cytokines such as Interleukin-1*β* (IL-1*β*), is recognized as a contributing factor to age-related cognitive impairments and muscle degeneration (51, 52). Moreover, hormonal changes with aging, like reduced growth hormone and testosterone, affect cognition and muscle health by decreasing protein synthesis and increasing muscle breakdown, leading to sarcopenia. Cognitive decline can worsen hormonal regulation, creating a feedback loop that intensifies both issues (53, 54). Antioxidant nutrients, dietary fats, and B vitamins are crucial for neurocognitive development and may help prevent sarcopenia by enhancing muscle function and minimizing oxidative stress (55).

In alignment with previous research, an elevated risk of impaired IC has been linked to insufficient HGS (56, 57). Sarcopenia, sharing diagnostic criteria with HGS deficiency, is strongly linked to vitality and locomotive capacity in older adults across various groups (58).

Aging, inflammation, oxidative stress, inactivity, and poor nutrition can affect the connection between locomotion and sarcopenia. As people age, muscle fibers, particularly fast-twitch ones, shrink and decline in number, reducing muscle strength and endurance. This diminishes the ability to perform daily tasks and may result in sarcopenia (59, 60). In addition, chronic inflammation, such as Tumor necrosis factor-alpha (TNF-*α*), leads to muscle catabolism and reduced muscle protein synthesis (61). Moreover, the imbalance between free radicals and antioxidants leads to oxidative stress, worsening muscle degradation and impairing function, which reduces locomotive capacity and raises the risk of sarcopenia (62, 63). Sedentary behavior can cause insulin resistance and muscle weakness, while regular locomotion enhances insulin sensitivity and muscle protein synthesis, mitigating the negative effects on muscle health (64, 65). Adequate protein intake is essential for muscle protein synthesis; insufficient protein can lead to muscle wasting and sarcopenia, especially in older adults. Protein supplementation, particularly with resistance training, may help preserve muscle mass in older adults (66, 67).

Sleep duration and quality as well as dietary intake have been identified as key factors affecting vitality and sarcopenia. Adequate sleep duration and quality may help improve vitality and mitigate the adverse effects of sarcopenia (68, 69). In addition, an adequate intake of energy and protein, particularly from animals and white meat, can help improve vitality and reduce bone loss in older adults, supporting muscle health (70, 71).

Sensory impairments, such as vision and hearing loss, can decrease physical activity by hindering navigation and social engagement. This inactivity is a known risk factor for sarcopenia, as it causes muscle wasting and weakened strength over time (72, 73). Vision and hearing impairment can affect an individual’s ability to prepare and consume a balanced diet, leading to inadequate nutrient intake. Deficiencies in protein and vitamin D, in particular, lead to muscle atrophy and weakness, which are key components of sarcopenia (74, 75).

Hormonal changes linked to depression, like increased cortisol, may contribute to sarcopenia by promoting muscle breakdown and inhibiting growth. Elevated cortisol, a stress hormone with catabolic effects, is often seen in depressed individuals and may lead to muscle degeneration (76, 77). Moreover, elevated inflammatory cytokines like interleukin-6 (IL-6) and TNF-*α* in depression patients may lead to muscle atrophy and weakness (78, 79).

The link between IC and TCBI in middle-aged and older individuals is intricate. Research conducted in China using an observational design identified an inverse relationship between cognitive decline and the TCBI among adults in midlife and older age groups (25). TG is a key part of TCBI, serving as the body’s main energy reserve. It breaks down into fatty acids and glycerol to fuel the brain, which is crucial for cognitive function, especially in older adults with decreased metabolic efficiency (80). Elevated levels of TC, which are essential for preserving cellular structure and function, have been linked to enhanced cognitive performance (81). Depression as part of the IC assessment of psychology is associated with TG. Depressed individuals often have reduced appetite and weight loss, leading to lower TG levels. Additionally, elevated stress hormones like cortisol in depression can alter lipid metabolism, further decreasing TG levels (82).

Mediation analyses revealed that TCBI partially mediated the association between IC and sarcopenia. Following comprehensive adjustment for covariates, each 1-unit elevation in TCBI corresponded to a 28% decline in sarcopenia risk (OR = 0.72, 95% CI: 0.58–0.90; *p* = 0.004), underscoring TCBI as a protective indicator of sarcopenia risk. The inverse relationship between TCBI and sarcopenia involves complex physiological mechanisms. TG is a key energy source that suggests sufficient energy reserves for muscle maintenance. Adequate energy levels are crucial for preserving muscle mass, as low energy can cause muscle breakdown and sarcopenia. Therefore, higher TG levels may indicate energy sufficiency, helping to prevent muscle loss (83). Furthermore, TG is essential for producing steroid hormones that are crucial for muscle protein synthesis and repair, helping to maintain muscle mass and prevent sarcopenia (84). Elevated TG levels might suggest improved insulin sensitivity, benefiting muscle health by enhancing glucose uptake, promoting muscle protein synthesis, and lowering sarcopenia risk (85, 86). Furthermore, sufficient TC levels support membrane fluidity and stability, crucial for muscle cell function and repair, helping preserve muscle mass and function in older adults (87). Elevated BMI is frequently linked to higher levels of circulating insulin and other anabolic hormones, which can enhance muscle protein synthesis and inhibit muscle breakdown. This anabolic state may aid in maintaining muscle mass in individuals with higher BMI, thereby explaining the inverse relationship with sarcopenia (88, 89).

In summary, public health strategies should integrate multidimensional IC assessments, including cognitive training, physical activity, and mental health support, into geriatric care. Nutritional interventions to optimize lipid profiles and manage weight through balanced diets and regular monitoring are crucial for improving TCBI. Policymakers should focus on community programs that encourage healthy aging through exercise, nutrition education, and early screenings. By enhancing functional capacity and optimizing metabolic health, we can reduce sarcopenia, ease healthcare burdens, and support healthy aging.

This investigation offers multiple advantages. Foremost, the utilization of longitudinal, population-based cohort data from China enhances the generalizability and empirical robustness of the results. Secondly, this investigation represents the inaugural effort to systematically examine the relationships between IC, TCBI, and sarcopenia risk within middle-aged and older populations. Finally, the potential mediator TCBI was evaluated, which further supports the mechanistic framework and provides a strong rationale for preventing and improving sarcopenia. Concurrently, this study acknowledges several limitations. Firstly, the research’s emphasis on middle-aged and older populations within China’s sociocultural and demographic context limits generalizability of findings to younger cohorts or diverse ethnic/cultural groups. Secondly, the cross-sectional survey design limits causal inference as it measures all variables simultaneously, preventing the establishment of time series and mechanistic pathways between exposures (e.g., IC, TCBI) and sarcopenia outcomes. It cannot determine bidirectional relationships, and TCBI might reflect metabolic adaptations to sarcopenia rather than being causal. Thirdly, unmeasured confounders such as chronic inflammation, physical activity, and dietary patterns could bias the associations and affect the magnitude of the mediating proportion. Fourthly, the exclusion of participants with missing data may have introduced selection bias, potentially limiting the generalizability of the findings. Lastly, this study’s reliance on existing data limited its scope, as it assessed the vitality domain using only BMI instead of the more widely recommended Mini Nutritional Assessment (MNA) scale by the WHO.

To address these limitations, longitudinal cohort studies that track IC, TCBI, and sarcopenia trajectories over time are essential for establishing temporal relationships and elucidating causal pathways. Intervention trials could further investigate whether enhancing IC (e.g., through multimodal exercise programs) or optimizing TCBI (e.g., via lipid-modulating therapies or nutritional interventions) reduces the incidence of sarcopenia. Mechanistic studies incorporating biomarkers (e.g., inflammatory cytokines, hormonal profiles) and advanced imaging techniques (e.g., DXA for muscle mass quantification) would strengthen the biological plausibility of observed associations. Additionally, validating TCBI’s predictive utility across diverse ethnic and clinical populations is critical to confirming its role as a universal nutritional indicator. Finally, integrating multidimensional assessments of vitality (e.g., combining BMI, dietary intake, and inflammatory markers) into IC frameworks could improve their sensitivity in detecting early functional decline. Such efforts would advance personalized strategies for sarcopenia prevention and promote healthy aging.

## Conclusion

The research revealed a notable negative association linking IC to sarcopenia, alongside a pronounced negative correlation between TCBI and sarcopenia. Mediation analysis identified TCBI as a partial intermediary in the pathway connecting IC to sarcopenia. These findings hold significant implications for healthcare policy and public health interventions designed to enhance the well-being of middle-aged and older populations affected by sarcopenia, through the assessment of functional and nutritional indicators.

## Data Availability

Publicly available datasets were analyzed in this study. This data can be found here: https://charls.pku.edu.cn/en/.
