# Supplementary material for: The relationship between intrinsic capacity and sarcopenia in middle-aged and older Chinese populations: the mediating influence of a novel nutritional index
Source: Front Public Health. 2025 May 21;13:1605158. doi: 10.3389/fpubh.2025.1605158 (PMC12133557; doi:10.3389/fpubh.2025.1605158)
Supplement: Supplementary file 1 [file Data_Sheet_1.zip › Supplementary Tables/Supplementary Table 1.doc]

Supplementary Table 1. Associations of TG,TC, and BW with sarcopenia.

| Variables | No | Unadjusted | | Model 1 | | Model 2 | | Model 3 | |
| --- | --- | --- | --- | --- | --- | --- | --- | --- | --- |
|  |  | OR(95% CI) | p value | OR (95% CI) | p value | OR(95% CI) | p value | OR (95% CI) | p value |
| TG | 6554 | 0.43(0.37~0.51) | <0.001 | 0.33(0.27~0.40) | <0.001 | 0.33(0.27~0.40) | <0.001 | 1.01 (0.78~1.31) | 0.95 |
| TC | 6554 | 1.16(0.79~1.71) | 0.44 | 0.44(0.28~0.69) | <0.001 | 0.42(0.27~0.66) | <0.001 | 0.85(0.46~1.58) | 0.62 |
| BW | 6554 | 0.62(0.60~0.64) | <0.001 | 0.62(0.60~0.65) | 0.001 | 0.62(0.60~0.65) | <0.001 | 0.68(0.65~0.71) | <0.001 |
| Model 1: adjusted for age, gender, educational level, marital status, and residence. Model 2: adjusted for model 1 + smoking status, drinking status, sleep duration and daytime napping duration. Model 3: adjusted for model 2 + BMI and 14 chronic diseases. Abbreviations: OR, odds ratio; 95% CI, 95% confidence interval; TG, Triglycerides, TC,Total Cholesterol, BW, Body Weight. | | | | | | | | | |
