# Supplementary material for: The relationship between intrinsic capacity and sarcopenia in middle-aged and older Chinese populations: the mediating influence of a novel nutritional index
Source: Front Public Health. 2025 May 21;13:1605158. doi: 10.3389/fpubh.2025.1605158 (PMC12133557; doi:10.3389/fpubh.2025.1605158)
Supplement: Supplementary file 1 [file Data_Sheet_1.zip › Supplementary Tables/Supplementary Table 2.doc]

Supplementary Table 2. Associations of IC with TG,TC, and BW.

| Variables | No | Unadjusted | | Model 1 | | Model 2 | | Model 3 | |
| --- | --- | --- | --- | --- | --- | --- | --- | --- | --- |
|  |  | β(95% CI) | p value | β(95% CI) | p value | β(95% CI) | p value | β (95% CI) | p value |
| TG | 6554 | 0.03(0.02~0.06) | <0.001 | 0.02(0.01~0.03) | 0.002 | 0.02 (0.01~0.03) | <0.001 | 0.03 (0.02~0.04) | <0.001 |
| TC | 6554 | 0.01(-0.01~0.02) | 0.26 | 0.01(0.01~0.02) | <0.001 | 0.01(0.01~0.02) | <0.001 | 0.01 (0.01~0.02) | <0.001 |
| BW | 6554 | 2.16(1.91~2.42) | <0.001 | 0.84(0.59~1.10) | <0.001 | 0.80(0.54~1.06) | <0.001 | 0.83(0.59~1.07) | <0.001 |
| Model 1: adjusted for age, gender, educational level, marital status, and residence. Model 2: adjusted for model 1 + smoking status, drinking status, sleep duration and daytime napping duration. Model 3: adjusted for model 2 + BMI and 14 chronic diseases. Abbreviations: 95% CI, 95% confidence interval; TG, Triglycerides, TC,Total Cholesterol, BW, Body Weight, IC, intrinsic capacity. | | | | | | | | | |
